# Do Tofacitinib Generics Exhibit Conventional Synthetic Disease-Modifying Anti-rheumatic Drug and Non-steroidal Anti-inflammatory Drug-Sparing Ability in the Management of Axial Spondyloarthritis?

**DOI:** 10.7759/cureus.46391

**Published:** 2023-10-02

**Authors:** Arvind Ganapati, Suvrat Arya, Nikhil Gupta, Abhishek Patil, Pramod Chebbi, Daisy Doley, Sachin R Jeevanagi, Rahul Sahu, Santosh K Mandal

**Affiliations:** 1 General Medicine/Rheumatology, Kasturba Medical College, Manipal, IND; 2 Rheumatology and Clinical Immunology, All India Institute of Medical Sciences, Mangalagiri, Guntur, IND; 3 Rheumatology, Jaypee Hospital, Noida, IND; 4 Rheumatology, Saroj Super Speciality Hospital, New Delhi, IND; 5 Rheumatology, Sant Parmanand Hospital, New Delhi, IND; 6 Rheumatology, Manipal Hospital, Bengaluru, IND; 7 Rheumatology, SDM (Shri Dharmasthala Manjunatheshwara) College of Medical Sciences and Hospital, SDM University, Dharwad, IND; 8 Rheumatology, Assam Medical College and Hospital, Dibrugarh, IND; 9 Rheumatology, Gurusharan Arthritis & Rheumatology Clinic, Kalaburagi, IND; 10 Rheumatology, People’s College of Medical Sciences & Research Centre, Bhopal, IND; 11 Rheumatology, Narayana Health, Rabindranath Tagore International Institute of Cardiac Sciences, Kolkata, IND

**Keywords:** sulfasalazine, methotrexate, nsaid, csdmard, asdas esr, clinically important improvement, axial spondyloarthritis, generics, tofacitinib, ankylosing spondylitis

## Abstract

Introduction: Tofacitinib has emerged as a therapeutic option for axial spondyloarthritis (axSpA) following successful clinical trials. The evidence on the efficacy of tofacitinib generics in the management of axSpA is limited. In India, the usage of conventional synthetic disease-modifying anti-rheumatic drugs (csDMARDs) is commonplace in the management of axSpA. Our primary aim was to identify the csDMARD and non-steroidal anti-inflammatory drug (NSAID)-sparing role of tofacitinib generics in an axSpA population.

Methods: This was a retrospective study in a real-world clinical setting. Data from nine rheumatology centers across India were analyzed for 168 patients with active axSpA who were initiated on generic tofacitinib 5 mg twice daily in conjunction with csDMARDs and NSAIDs, over a duration of six months. Our primary outcome was to evaluate the csDMARD and NSAID-sparing effect of tofacitinib generics, while the secondary outcome assessed safety profiles and efficacy at six months.

Results: The median Ankylosing Spondylitis Disease Activity Score (ASDAS) erythrocyte sedimentation rate (ESR) score of the study population was 3.91 (3.26, 4.56). Alongside tofacitinib generics, 121 (72%) patients were co-administered csDMARDs (methotrexate/sulfasalazine/both), and 90 (53.6%) patients were co-administered NSAIDs. The csDMARD, NSAID, and combined csDMARD + NSAID-sparing effects of tofacitinib generics were seen in 85 (50.6%), 156 (92.9%), and 81 (48.2%) patients, respectively. Adverse events were mild and well-tolerated. At six months, 124 (57.9%) patients had attained clinically important improvement in ASDAS ESR score, and the median decrease in ASDAS ESR score was 2.02 (1.18, 2.96).

Conclusion: This real-world study provides evidence supporting the csDMARD and NSAID-sparing ability of tofacitinib generics in the treatment of axSpA. Tofacitinib generics displayed a good safety profile and showed signals of efficacy as well.

## Introduction

Axial spondyloarthritis (axSpA) is a chronic inflammatory disorder. It primarily affects the sacroiliac joints and the axial skeleton. If left untreated, it can even lead to progressive stiffness and deformity of the axial skeleton in some patients. Additionally, it can cause inflammatory involvement of other peripheral joints, entheses, uveal tissue, bowel, etc. [1].

Pharmacotherapy of the disease primarily includes non-steroidal anti-inflammatory drugs (NSAIDs) and biological agents (tumor necrosis factor inhibitors and IL-17 inhibitors), with a limited role of conventional synthetic disease-modifying anti-rheumatic drugs (csDMARDs) [2]. However, in recent years, Janus kinase (JAK) inhibitors like upadacitinib, tofacitinib, and filgotinib have emerged as an addition to the therapeutic armamentarium of axSpA, following their success in clinical trials [3-7]. The JAK/STAT pathway plays a critical role in the signaling of the IL-23/IL-17 axis, which is a significant contributor to axSpA pathogenesis [8]. TYK2, JAK2, and JAK1 serve as essential therapeutic targets in axSpA. Tofacitinib primarily targets JAK1 and JAK3, with lesser effects on JAK2 [9]. It showed promising results in phase II and phase III clinical trials [5,6]. Subsequently, tofacitinib was approved by the United States Food and Drug Administration in 2021 [10] and the European Union for the management of axSpA [11], and has been adapted into the 2022 update of the Assessment of Spondyloarthritis International Society (ASAS)-European Alliance of Associations for Rheumatology (EULAR) recommendations for the management of axSpA [12]. Tofacitinib therapy has also shown improvement in other domains of axSpA, such as fatigue, health-related quality of life, indices of work productivity [13], and MRI-based improvement in inflammation [14].

Being an oral agent, tofacitinib use is convenient for patients and not associated with anti-drug antibodies like those seen with biological agents. Generics of tofacitinib are widely accessible in India, providing substantial cost reductions and reducing monthly therapy expenses to 1/10th of the original molecule's cost. Evidence on its efficacy is scarce, but a recent, small-scale, single-center, retrospective study from India, published in 2023, shows that tofacitinib generics significantly reduced the Ankylosing Spondylitis Disease Activity Score (ASDAS) C-reactive protein (CRP) score in patients and demonstrated a good safety profile [15]. However, no study has evaluated the NSAID and disease-modifying anti-rheumatic drug (DMARD)-sparing ability of tofacitinib generics in the therapy of axSpA to date. The use of csDMARDs in the treatment of axSpA is common in India, even though high-quality evidence for efficacy and validation in ASAS-EULAR guidelines for axSpA management are lacking [12]. The evidence of the efficacy of csDMARDs in axSpA has not been proven in high-quality clinical studies, but only in low-quality randomized trials or observational studies of a small scale [16-19].

The purpose of this study was to evaluate the csDMARD and NSAID-sparing capability of generics of tofacitinib in treating axSpA in a real-world clinical setting.

## Materials and methods

Study design and research setting

The study was a retrospective study of prospectively collected data, conducted at nine rheumatology centers across India, including three teaching hospitals. The study was approved by the institutional review boards of the respective institutes. A waiver of consent was approved due to the retrospective nature of the study.

Inclusion criteria

Patients over 18 years of age were recruited, satisfying the ASAS classification of axSpA [20] with active disease (as per ASDAS erythrocyte sedimentation rate (ESR) or CRP ≥ 2.1) [21], and initiated on tofacitinib generics at a dosage of 5 mg twice daily as per the discretion of treating rheumatologist. Further, only patients who maintained adherence and compliance to the same dosage for a minimum of six months were included.

Study time frame and duration of follow-up

The study recruitment was carried out from January 2022 to June 2022. The follow-up details of these patients were recorded for a period of six months from the date of initiation (not beyond January 2023).

Patient variables assessed at baseline

Demographic characteristics, clinical history, investigation details, treatment details, including csDMARD and NSAID intake, visual analog scale (VAS) spinal pain, VAS peripheral joint pain, early morning stiffness in the spine (questions 2, 3, 5, and 6 of Bath Ankylosing Spondylitis Disease Activity Index (BASDAI)) [22], Bath Ankylosing Spondylitis Patient Global Score (BAS-G) [23], and disease and activity scores like ASDAS ESR were recorded from the electronic medical records of patients from their out-patient visits.

Patient variables assessed at three-month and six-month follow-ups

Details pertaining to compliance, adverse events, NSAID intake, csDMARDs, generic tofacitinib, ESR, ASDAS ESR, pain, and stiffness-related questions 2, 3, 5, and 6 of BASDAI, and BAS-G over the six-month period were also recorded. Additionally, clinically important improvement (CII) and major improvement (MI) in ASDAS ESR scores [21] on follow-up were calculated from the recorded data, and a switch to biologicals was also noted.

Study definitions

csDMARD Spared

During the study period, if there was (1) complete cessation of csDMARD, (2) dose reduction of any csDMARD administered from baseline, (3) a reduction in the number of csDMARD prescribed from baseline, and (4) no new initiation of csDMARD in patients who were not offered any at baseline, then the patients were categorized as csDMARD-spared.

csDMARD Non-spared

During the study period, if there was (1) a new introduction of csDMARD in patients who were not offered any csDMARD at baseline, (2) a dose increment of any csDMARD administered from baseline, (3) an increase in the number of csDMARD prescribed from baseline, (4) maintenance of the same dose of csDMARD prescribed at baseline throughout the study, and (5) a change of one csDMARD from baseline to another csDMARD, then the patients were categorized as csDMARD non-spared.

NSAID Spared

During the study period, if there was (1) complete cessation of NSAIDs, (2) dose reduction of NSAID assessed on the Likert scale (25%, 50%, and 75% reduction from the scale), and (3) no initiation of NSAID in patients who were not offered any NSAIDs at baseline, then the patients were categorized as NSAID spared.

NSAID Non-spared

During the study period, if there was (1) continuation of NSAIDs at the same dose as baseline use and (2) worse NSAID intake compared to baseline use (both assessed on the Likert scale), then the patients were categorized as NSAID non-spared.

Outcomes assessed

The primary outcome assessed was the csDMARD and NSAID-sparing ability (using the pre-specified study definition) of generic tofacitinib in axSpA over a six-month treatment period and predictors for the same. The secondary outcome assessed was safety profile and efficacy parameters at six months in the study population.

Statistical methods

Continuous variables are represented as median (with interquartile range) after verifying the normality of the data distribution. Categorical variable data are represented in frequencies/percentages. A comparison of categorical variables with the primary outcome was performed using the chi-square test. A comparison of continuous variables with the primary outcome was done with the Mann-Whitney U test. A p-value of <0.05 was considered statistically significant. The outcome measures recorded at three time points were compared using univariate ANOVA for repeated measures. Multivariate logistic regression was performed on data parameters with p-value < 0.1. Statistical analysis was performed using SPSS version 16 (IBM Corp., Armonk, NY).

## Results

A total of 168 patients who were initiated on tofacitinib generics 5 mg twice daily and maintained on the same for six months were identified. Baseline characteristics of the patients have been highlighted in Table 1.

**Table 1:** Baseline characteristics of the study population IQR, interquartile range; HLA, human leukocyte antigen; VAS, visual analog scale; BASDAI, Bath Ankylosing Spondylitis Disease Activity Index; BAS-G, Bath Ankylosing Spondylitis Patient Global Score; ASDAS, Ankylosing Spondylitis Disease Activity Score; NSAID, non-steroidal anti-inflammatory drug; ESR, erythrocyte sedimentation rate; CRP, C-reactive protein; csDMARD, conventional synthetic disease-modifying anti-rheumatic drug.

Baseline characteristics	Value
Age in years, median with IQR	35 (27, 43)
Male-to-female ratio (n:n)	116:52
Duration of disease in months, median with IQR	60 (36, 120)
Type of disease, n (%)	Axial only involvement: 74 (44.0)
	Axial and peripheral involvement: 94 (56.0)
Radiographic; non-radiographic disease, n (%); n (%)	154 (91.7); 14 (8.3)
HLA B27 status, n (%)	Positive: 131 (78.0)
	Negative: 22 (13.1)
	Not done: 15 (8.9)
Type of axial spondyloarthritis (based on etiology), n (%)	Primary: 145 (86.3)
	Psoriatic: 10 (6.0)
	Undifferentiated spondyloarthritis: 6 (3.6)
	Enteropathic: 7 (4.1)
Other manifestations of disease, n (%)	Enthesitis: 86 (51.2)
	Uveitis: 42 (25.0)
	Dactylitis: 15 (8.9)
Biological use within the last 6 months, n (%)	4 (2.4)
Treatment received within 3 months prior to initiation of the study, n (%)	NSAID only: 17 (10.1)
	Methotrexate only: 6 (3.6)
	Methotrexate + NSAID: 2 (1.2)
	Sulfasalazine only: 17(10.6)
	Sulfasalazine + NSAID: 45 (26.8)
	Methotrexate + sulfasalazine: 9 (5.3)
	Methotrexate + sulfasalazine + NSAID: 67 (39.9)
	Sulfasalazine + leflunomide + NSAID: 1 (0.6)
	Methotrexate + sulfasalazine + leflunomide + NSAID: 1 (0.6)
	None: 6 (3.6)
VAS spinal pain (question 2, BASDAI), median with IQR	6 (5, 8)
VAS pain peripheral joints (question 3, BASDAI), median with IQR	1 (0, 6)
Early morning stiffness duration (spine) in minutes, median with IQR	60 (30, 75)
BAS-G, median with IQR	6 (5, 8)
ESR in mm/1^st^ hour, median with IQR	54 (35, 71)
ASDAS ESR, median with IQR	3.91 (3.26, 4.56)
ASDAS CRP (n = 167), median with IQR	4.39 (3.52, 5.58)
Tuberculosis pre-screening, n (%)	Tuberculin skin test: 99 (58.9)
	Interferon-gamma release assay: 109 (64.9)
	Chest X-ray: 161 (95.8)
	High-resolution CT thorax: 9 (5.4)
	None: 3 (1.8)
Co-treatment initiated with tofacitinib, n (%)	NSAID only: 38 (22.6)
	Methotrexate only: 17 (10.1)
	Methotrexate + NSAID: 11 (6.5)
	Sulfasalazine only: 22 (13.1)
	Sulfasalazine + NSAID: 28 (16.7)
	Methotrexate + sulfasalazine: 30 (17.8)
	Methotrexate + sulfasalazine + NSAID: 13 (7.7)
	None: 8 (4.8)
Dose of csDMARD, n	Methotrexate 15 mg/week: 61
	Methotrexate 10 mg/week: 10
	Sulfasalazine (2 gm/day): 93

Primary outcome

csDMARD and NSAID-Sparing Effect of Generic Tofacitinib Over Six Months

The csDMARD-sparing effect of tofacitinib generics was seen in 85 (50.6%) patients and the NSAID-sparing effect was seen in 156 (92.9%) patients. The median time to dose reduction/cessation of csDMARDs was 90 (90, 105) days. Out of the 71 patients who were on methotrexate, dose reduction was achieved in 18 (25.4%) patients, and discontinuation of methotrexate was observed in 11 (15.5%) patients. Out of the 93 patients who were on sulfasalazine, dose reduction was achieved in four (4.3%) patients, and cessation of sulfasalazine administration was seen in 16 (17.6%) patients. There was no introduction of csDMARD over the six-month period in 40 (23.8%) patients who were never initiated on csDMARD at baseline. csDMARD change-over was noted in two (1.2%) patients, the continuation of the same dose and the same number of csDMARD was seen in 71 (42.3%) patients, and the addition of new csDMARD was noted in 10 (5.9%) patients. Out of 168 patients, combined csDMARD and NSAID-sparing effect was seen in 81 (48.2%) patients. At six months, no NSAID intake was observed in 119 (70.8%) patients, with less than 25% intake from baseline in seven (4.2%) patients, less than 50% from baseline in seven (4.2%) patients, and less than 75% from baseline in 23 (13.7%) patients. In nine (5.3%) patients, the NSAID intake remained the same as baseline usage and was worse than baseline in three (1.8%) patients.

Factors Predicting csDMARD and NSAID-Sparing Effect at Six Months

Univariate analysis revealed that parameters at baseline like median early morning stiffness in the spine, median ESR, median ASDAS ESR, absence of enthesitis, and CII response at three and six months were significantly associated with the csDMARD-sparing effect of tofacitinib generics (p-value < 0.05) (Table 2). However, multivariate analysis showed that only the absence of enthesitis at baseline and CII response attainment at six months predicted a csDMARD-sparing effect (p-value = 0.002). Univariate analysis revealed that CII responses at three and six months were significantly associated with the NSAID-sparing effect of tofacitinib generics (p-value < 0.05) (Table 3). However, no predictors were identified in multivariate analysis for this effect. Univariate analysis revealed that baseline parameters like median early morning stiffness in the spine, median ASDAS ESR, absence of enthesitis, and CII response at three and six months were significantly associated with the combined csDMARD and NSAID-sparing effect of tofacitinib generics (p-value < 0.05) (Table 4). However, multivariate analysis showed that only the attainment of CII response at six months predicted the occurrence of a combined csDMARD and NSAID-sparing effect (p-value = 0.001) (Tables 5, 6).

**Table 2:** Comparison of parameters between csDMARD-spared and non-spared patients by univariate analysis csDMARD, conventional synthetic disease-modifying anti-rheumatic drug; HLA, human leukocyte antigen; VAS, visual analog scale; EMS, early morning stiffness; BAS-G, Bath Ankylosing Spondylitis Patient Global Score; ESR, erythrocyte sedimentation rate; ASDAS, Ankylosing Spondylitis Disease Activity Score; AS, ankylosing spondylitis; NSAID, non-steroidal anti-inflammatory drug; CII, clinically important improvement in ASDAS score.

Parameters	csDMARD spared (n = 85)	csDMARD non-spared (n = 83)	P-value
Male gender (%)	69.4	68.7	0.9
Median age, baseline (years)	35.1	35.4	0.9
Median duration of disease, baseline (months)	60	60	0.5
HLA B27 positivity (%)	87.6	83.3	0.4
Median VAS spinal pain, baseline	6.6	6.2	0.2
Median VAS peripheral joint pain, baseline	3.4	2.7	0.1
Median EMS, baseline	62.8	52.5	0.01
Median BAS-G, baseline	6.5	6.4	0.9
Median ESR, baseline (mm/1st hour)	57.8	50.2	0.04
Median ASDAS ESR, baseline	4.1	3.72	0.02
Presence of axial manifestations (%)	50.6	37.3	0.08
Primary AS etiology (%)	90.5	81.9	0.1
Presence of enthesitis, baseline (%)	40.0	62.6	0.003
Presence of uveitis, baseline (%)	27	21.7	0.4
Presence of dactylitis, baseline (%)	11.8	6.0	0.2
NSAID users, baseline (%)	78.8	79.5	0.9
CII responders at 3 months (%)	73.5	59.0	0.05
CII responders at 6 months (%)	84.7	62.5	0.001

**Table 3:** Comparison of parameters between NSAID-spared and non-spared patients by univariate analysis csDMARD, conventional synthetic disease-modifying anti-rheumatic drug; HLA, human leukocyte antigen; VAS, visual analog scale; EMS, early morning stiffness; BAS-G, Bath Ankylosing Spondylitis Patient Global Score; ESR, erythrocyte sedimentation rate; ASDAS, Ankylosing Spondylitis Disease Activity Score; NSAID, non-steroidal anti-inflammatory drug; CII, clinically important improvement in ASDAS score.

Parameters	NSAID spared (n = 156)	NSAID non-spared (n = 12)	P-value
Male gender (%)	68.0	83.3	0.4
Median age, baseline (years)	35.7	35.2	0.9
Median duration of disease, baseline (months)	89.2	48.9	0.08
HLA B27 positivity (%)	86.0	80.0	0.9
Median VAS spinal pain, baseline	6.4	6.7	0.6
Median VAS peripheral joint pain, baseline	3.2	1.5	0.2
Median EMS, baseline	57.1	65.8	0.2
Median BAS-G, baseline	6.0	6.5	0.4
Median ESR, baseline (mm/1st hour)	54.4	48.8	0.7
Median ASDAS ESR, baseline	3.9	3.7	0.4
Presence of axial manifestations (%)	42.3	66.7	0.2
Primary AS etiology (%)	85.2	91.6	0.8
Presence of enthesitis, baseline (%)	51.9	41.7	0.1
Presence of uveitis, baseline (%)	24.4	33.3	0.8
csDMARD users, baseline (%)	73.1	58.3	0.4
CII responders at 3 months (%)	68.9	33.3	0.02
CII responders at 6 months (%)	78.2	16.7	0.001

**Table 4:** Comparison of parameters between combined csDMARD + NSAID-spared and non-spared patients by univariate analysis csDMARD, conventional synthetic disease-modifying anti-rheumatic drug; NSAID, non-steroidal anti-inflammatory drug; HLA, human leukocyte antigen; VAS, visual analog scale; EMS, early morning stiffness; BAS-G, Bath Ankylosing Spondylitis Patient Global Score; ESR, erythrocyte sedimentation rate; ASDAS, Ankylosing Spondylitis Disease Activity Score; AS, ankylosing spondylitis; CII, clinically important improvement in ASDAS score.

Parameters	csDMARD + NSAID spared (n = 81)	csDMARD + NSAID non-spared (n = 87)	P-value
Male gender (%)	67.9	70.1	0.7
Median age, baseline (years)	34.9	35.6	0.7
Median duration of disease, baseline (months)	82.9	90.4	0.7
HLA B27 positivity (%)	88.3	82.9	0.3
Median VAS spinal pain, baseline	6.6	6.3	0.2
Median VAS peripheral joint pain, baseline	3.5	2.6	0.2
Median EMS, baseline	62.3	53.5	0.03
Median BAS-G, baseline	6.5	6.4	0.6
Median ESR, baseline (mm/1st hour)	57.5	50.7	0.08
Median ASDAS ESR, baseline	4.1	3.9	0.02
Presence of axial disease, baseline (%)	49.4	39.1	0.2
Primary AS, etiology (%)	90.1	82.8	0.2
Presence of enthesitis, baseline (%)	40.7	60.9	0.01
Presence of uveitis, baseline (%)	25.9	23	0.6
Presence of dactylitis, baseline (%)	12.2	5.8	0.1
CII responders at 3 months (%)	75.9	57.3	0.01
CII responders at 6 months (%)	87.6	60.9	0.001

**Table 5:** Multivariate regression analysis for predictors of csDMARD-sparing effects of tofacitinib generics csDMARD, conventional synthetic disease-modifying anti-rheumatic drug; EMS, early morning stiffness; ESR, erythrocyte sedimentation rate; ASDAS, Ankylosing Spondylitis Disease Activity Score; CII, clinically important improvement in ASDAS score.

Parameter	Estimate	Standard error	T statistic	P-value
Constant	0.375	0.213	1.762	0.08
Median EMS in the spine, baseline	0.0009	0.001	0.519	0.6
Median ESR, baseline	-0.0001	0.002	-0.052	0.9
Median ASDAS ESR, baseline	0.027	0.094	0.287	0.7
Absence of enthesitis, baseline	-0.178	0.079	-2.250	0.02
CII at 3 months	0.037	0.094	0.395	0.7
CII at 6 months	0.231	0.09	2.384	0.01
Presence of axial symptoms	-0.080	0.086	-0.927	0.3
				0.002 (overall)

**Table 6:** Multivariate regression analysis for predictors of csDMARD + NSAID-sparing effects of tofacitinib generics csDMARD, conventional synthetic disease-modifying anti-rheumatic drug; NSAID, non-steroidal anti-inflammatory drug; EMS, early morning stiffness; ESR, erythrocyte sedimentation rate; ASDAS, Ankylosing Spondylitis Disease Activity Score; CII, clinically important improvement in ASDAS score.

Parameter	Estimate	Standard error	T statistic	P-value
Constant	0.286	0.211	1.353	0.1
Median EMS in the spine, baseline	0.0002	0.001	0.114	0.9
Median ESR, baseline	-0.001	0.002	-0.51	0.6
Median ASDAS ESR, baseline	0.05	0.093	0.544	0.6
Absence of enthesitis, baseline	-0.154	0.078	-1.962	0.05
CII at 3 months	0.071	0.093	0.761	0.4
CII at 6 months	0.289	0.096	3.001	0.003
Presence of axial symptoms	-0.077	0.085	-0.901	0.3
				0.001 (overall)

Secondary outcomes

Efficacy

At six months, 124 (57.9%) axSpA patients achieved CII, and the median decline in ASDAS ESR score was 2.02 (1.18, 2.96), with tofacitinib generics therapy in conjunction with csDMARD and NSAID usage. Three patients had escaped to biologicals and were considered as CII non-responders and none of these had DMARD-sparing or NSAID-sparing effect seen with tofacitinib generics. Other efficacy outcome parameters and changes at two follow-up visits from baseline are depicted in Tables 7, 8.

**Table 7:** Secondary outcome parameters, differences in various disease activity measures, and concomitant drug usage from baseline to three-month and six-month follow-ups in the study population IQR, interquartile range; VAS, visual analog scale; BASDAI, Bath Ankylosing Spondylitis Disease Activity Index; EMS, early morning stiffness; BAS-G, Bath Ankylosing Spondylitis Patient Global Score; ASDAS, Ankylosing Spondylitis Disease Activity Score; NSAID, non-steroidal anti-inflammatory drug; csDMARD, conventional synthetic disease-modifying anti-rheumatic drugs; ESR, erythrocyte sedimentation rate; CRP, C-reactive protein.

Parameter	Value at 3 months (n = 161)	Value at 6 months (n = 168)
Clinically important improvement in ASDAS ESR, n (%)	107 (63.7)	124 (57.9)
Major improvement in ASDAS ESR, n (%)	55 (32.7)	85 (50.6)
∆ VAS spinal pain (BASDAI question 2), median with IQR	3 (2, 5)	5 (3, 6)
∆ VAS peripheral joint pain (BASDAI question 3), median with IQR	0 (0, 4)	0 (0, 5)
∆ EMS in the spine in minutes, median with IQR	30 (15, 60)	40 (25, 60)
∆ BAS-G, median with IQR	3 (2, 4.5)	5 (3, 6)
∆ ESR in mm/1^st^ hour, median with IQR	24 (8, 44)	30 (7, 50)
∆ ASDAS ESR, median with IQR	1.63 (0.8, 2.51)	2.02 (1.18, 2.96)
NSAID usage, n (%)	No intake: 86 (53.4)	No intake: 119 (70.8)
	Less than 25% from baseline: 12 (7.5)	Less than 25% from baseline: 7 (4.2)
	Less than 50% from baseline: 16 (9.9)	Less than 50% from baseline: 7 (4.2)
	Less than 75% from baseline: 39 (24.2)	Less than 75% from baseline: 23 (13.7)
	Same as baseline: 5 (3.1)	Same as baseline: 9 (5.3)
	Worse than baseline: 3 (1.9)	Worse than baseline: 3 (1.8)
Co-treatment usage, n (%)	NSAID only: 21 (13.0)	NSAID only: 18 (10.7)
	Methotrexate only: 23 (14.3)	Methotrexate only: 23 (13.7)
	Methotrexate + NSAID: 3 (1.9)	Methotrexate + NSAID: 3 (1.8)
	Sulfasalazine only: 33 (20.5)	Sulfasalazine only: 35 (20.9)
	Sulfasalazine + NSAID: 10 (6.2)	Sulfasalazine + NSAID: 8 (4.8)
	Methotrexate + sulfasalazine: 33 (20.5)	Methotrexate + sulfasalazine: 30 (17.9)
	Methotrexate + sulfasalazine + NSAID: 5 (3.1)	Methotrexate + sulfasalazine + NSAID: 4 (2.4)
	None: 33 (20.5)	None: 47 (28.0)
Dose of csDMARD, n	Methotrexate (15 mg/week): 56	Methotrexate (15 mg/week): 53
	Methotrexate (10 mg/week): 8	Methotrexate (10 mg/week): 7
	Sulfasalazine (2 gm/day): 78	Sulfasalazine (2 gm/day): 73
	Sulfasalazine (1 gm/day): 3	Sulfasalazine (1 gm/day): 4

**Table 8:** Changes in various disease activity parameters from baseline to six months, assessed with univariate ANOVA for repeated measures SD, standard deviation; VAS, visual analog scale; EMS, early morning stiffness; BAS-G, Bath Ankylosing Spondylitis Patient Global Score; ASDAS, Ankylosing Spondylitis Disease Activity Score; ESR, erythrocyte sedimentation rate.

Parameter	Baseline	3 months	6 months	P-value
VAS spinal pain, mean± SD	6.5 ± 1.9	2.8 ± 2.0	1.8 ± 1.8	0.001
VAS peripheral joint pain, mean ± SD	3.0 ± 3.4	1.0 ± 1.5	0.6 ± 1.2	0.001
EMS in the spine duration (minutes), mean ± SD	57.3 ± 28.8	19.9 ± 21.3	13.8 ± 21.2	0.001
BAS-G, mean ± SD	6.4 ± 1.7	3.0 ± 1.9	1.9 ± 1.7	0.001
ESR(mm/1^st^ hour), mean ± SD	54.7 ± 27.5	28.5 ± 19.1	25.4 ± 21.2	0.001
ASDAS ESR, mean ± SD	3.9 ± 0.9	2.2 ± 0.8	1.8 ± 0.9	0.001

Adverse Events

At three-month follow-up, the most common adverse event was headache (n = 3), followed by herpes zoster, sleep deprivation, anxiety, and decreased appetite (n = 2 each). Weight gain, upper respiratory tract infection, urinary tract infection, abdominal pain, and lobar pneumonia were encountered in one patient each. At six-month follow-up, the most common adverse event noted was weight gain (n = 8), followed by urinary tract infection, upper respiratory infection, and herpes zoster (n = 2 each). Headache, abdominal pain, weight loss, sleep deprivation, and dyslipidemia were encountered in one patient each. No case of tuberculosis, deep vein thrombosis, or adverse cardiovascular event was observed in the study period. In the patient with lobar pneumonia and one patient with herpes zoster within the first three months, there was temporary discontinuation of tofacitinib generics, with re-initiation following recovery.

## Discussion

In this real-life, multi-center, clinical setting retrospective study, where the csDMARD and NSAID-sparing ability of tofacitinib generics was assessed in 168 active axSpA patients on background NSAID and csDMARD therapy, it was observed that tofacitinib generics contributed to 50% csDMARD-sparing effect, 93% NSAID-sparing effect, and 48% combined csDMARD and NSAID-sparing effect. This effect has not been documented in any previous study involving tofacitinib generics or the originator of tofacitinib in axSpA. This is the novelty of this study. These findings could be relevant to the South Asian countries where csDMARD usage (both methotrexate and sulfasalazine) in purely axial disease is rampant in real-life clinical practice despite poor efficacy and poor quality of evidence supporting this. The reason for this could be the inhibitory cost of biological/biosimilar agents or the tofacitinib originator, and the lack of other cheaper, efficacious agents. The evidence presented in our study could help cut down the csDMARD usage in purely axSpA, as tofacitinib generics provide an alternative. Also, tofacitinib may help achieve an NSAID-sparing effect, which is always desirable in axSpA, due to adverse events associated with their long-term usage.

Multivariate analysis revealed that only the absence of enthesitis at baseline and the attainment of a CII response at six months were independently and significantly associated with the csDMARD-sparing effect. This suggests that the presence of enthesitis makes the clinicians continue to use csDMARDs in addition to tofacitinib generics and attainment of CII response helps them discontinue csDMARDs. There was a 41% reduction in methotrexate usage compared to a 22% reduction in sulfasalazine usage. This implies that tofacitinib generics help clinicians align with ASAS-EULAR recommendations for the management of axSpA, wherein there is no scope for methotrexate usage [12]. Multivariate analysis showed that attainment of CII response at six months independently predicted combined csDMARD and NSAID-sparing effect, suggesting that the efficacy of tofacitinib generics helps achieve this effect.

In a recent study by Phatak et al. from India, the efficacy and safety of tofacitinib generics were demonstrated in a real-world clinical setting. A total of 100 patients of axSpA were treated with tofacitinib generic monotherapy; a mean improvement of 1.07 by ASDAS-CRP score was reported at a median follow-up duration of 192 days [15]. In our study, an improvement in disease activity assessed by median ∆ ASDAS ESR scores from baseline to six months was 2.02. In the study by Phatak et al., the recruited patient number was smaller, there was no background csDMARD usage, and no reporting of NSAID usage. Our study had a larger study population, and information on NSAID intake has been presented as an outcome parameter.

In a phase II clinical trial of tofacitinib in ankylosing spondylitis (AS) with the 5 mg twice-daily dosage schedule, CII attainment at 12 weeks was seen in 63.5% of patients and the ∆ ASDAS score was -1.4 [5]. In a phase III clinical trial of tofacitinib in AS in the 5 mg twice-daily dosage schedule, the CII at 16 weeks was seen in 61.4% and the ∆ ASDAS score was -1.39 [6]. In our study, 63.7% of patients attained CII at three months and the ∆ ASDAS score was -1.63, which is comparable to the evidence in the literature. Furthermore, we observed CII attainment rate at six months was 57.9% and the ∆ ASDAS score was -2.02. Since the current study had background csDMARD and NSAID intake, which could be confounding factors, we cannot overemphasize the efficacy of the data. The study results cannot be disregarded as the axSpA patients entering the study had active disease with a median ASDAS ESR score of 3.91, despite background csDMARD usage of 61.3% and NSAID usage of 79.2%. Hence, tofacitinib generics could have impacted the reduction of ASDAS ESR score and CII attainment, suggesting efficacy signals. From our results, we infer, that the evaluation of efficacy/non-inferiority of tofacitinib generics would be best assessed by utilizing a randomized controlled trial design with tofacitinib originator molecule/biological agent as the active comparator. The retrospective design, relatively small sample size, and lack of a comparator are weaknesses in the current study.

As indicated by Phatak et al. [15], the adverse events with tofacitinib generics in our study also were mostly mild and did not lead to complete cessation of tofacitinib generics therapy. A temporary cessation of treatment was seen in one patient with lobar pneumonia and one patient with herpes zoster within the first three months of assessment. However, following recovery from infection, they were re-initiated on tofacitinib generics. The observation is in line with the established safety profile of tofacitinib in the literature [24]. No case of tuberculosis, deep vein thromboses (DVT), or adverse cardiovascular events was noted in any of the patients during the study period. The herpes zoster infection rate during this study period was 4.76 per 100 patient years, consistent with reported literature in rheumatoid arthritis and ulcerative colitis during tofacitinib therapy [25]. Data on herpes zoster infection rates in spondyloarthritis are limited to one phase II (n = 2) and one phase III trial (n = 5) [5,6]. Interestingly, there were no cases of herpes zoster noted in the study by Phatak et al. [15]. However, real-life studies and phase IV studies with bigger sample sizes and long-term follow-up are needed to identify the tuberculosis risk, DVT, cardiovascular events, and malignancies with tofacitinib generic therapy in India and other developing world.

## Conclusions

The study reports findings regarding the csDMARD and NSAID-sparing effect, safety, and efficacy with the use of generic tofacitinib in axSpA treatment The study provides evidence of a significant sparing effect on NSAIDs and csDMARDs when patients were treated with generic tofacitinib. The majority of patients experienced no major safety concerns while undergoing therapy, with only minor adverse events reported. Generic tofacitinib has demonstrated efficacy signals in various disease activity parameters of axSpA in a substantial number of patients, warranting further analysis in larger randomized clinical trials. These findings could potentially modify the management strategies of axSpA in Indian subcontinents, especially where the drug cost and availability could affect treatment decisions.
